# Dynamic Bayesian network for predicting physiological changes, organ dysfunctions and mortality risk in critical trauma patients

**DOI:** 10.1186/s12911-022-01803-y

**Published:** 2022-05-03

**Authors:** Qi Chen, Bihan Tang, Jiaqi Song, Ying Jiang, Xinxin Zhao, Yiming Ruan, Fangjie Zhao, Guosheng Wu, Tao Chen, Jia He

**Affiliations:** 1 Department of Health Statistics, Naval Medical University, No. 800 Xiangyin Road, Shanghai, 200433 China; 2 School of Medicine, Tongji University, Shanghai, China; 3 Institute of Military Health Management, Naval Medical University, Shanghai, China; 4 Department of Cardiology, PLA General Hospital, No. 28 Fuxing Road, Beijing, 100853 China; 5 Department of Burn Surgery, Changhai Hospital, Naval Medical University, No. 800 Xiangyin Road, Shanghai, 200433 China; 6 Department of Pharmaceutical Administration and Regulation, Zhejiang Pharmaceutical College, Ningbo, China

**Keywords:** Dynamic Bayesian network, Critical trauma patients, Prediction model

## Abstract

**Background:**

Critical trauma patients are particularly prone to increased mortality risk; hence, an accurate prediction of their conditions enables early identification of patients' mortality status. Thus, we aimed to develop and validate a real-time prediction model for physiological changes, organ dysfunctions and mortality risk in critical trauma patients.

**Methods:**

We used Dynamic Bayesian Networks (DBNs) to model complicated relationships of physiological variables across time slices, accessing data of trauma patients from the Medical Information Mart for Intensive Care database (MIMIC-III) (n = 2915) and validated with patients' data from ICU admissions at the Changhai Hospital (ICU-CH) (n = 1909). The DBN model's evaluation included the predictive ability of physiological changes, organ dysfunctions and mortality risk.

**Results:**

Our DBN model included two static variables (age and sex) and 18 dynamic physiological variables. The differences in ratios between the real values and the 24- and 48-h predicted values of most physiological variables were within 5% in the two datasets. The accuracy of our DBN model for predicting renal, hepatic, cardiovascular and hematologic dysfunctions was more than 0.8.The calculated area under the curve (AUC) from receiver operating characteristic curves and 95% confidence interval for predicting the 24- and 48-h mortality risk were 0.977 (0.967–0.988) and 0.958 (0.945–0.971) in the MIMIC-III and 0.967 (0.947–0.987) and 0.946 (0.925–0.967) in ICU-CH.

**Conclusions:**

A DBN is a promising method for predicting medical temporal data such as trauma patients' mortality risk, demonstrated by high AUC scores and validation by a real-life ICU scenario; thus, our DBN prediction model can be used as a real-time tool to predict physiological changes, organ dysfunctions and mortality risk during ICU admissions.

**Supplementary Information:**

The online version contains supplementary material available at 10.1186/s12911-022-01803-y.

## Background

Trauma is a universal health challenge that leads to numerous deaths and disabilities at any age [1]. The common causes of trauma include road injuries, falls, self-harm, interpersonal violence, and so on. In 2017, there were more than 4.4 million trauma deaths and 520 million trauma cases globally, which resulted in 3267 DALYs per 100,000 [2]. Critical trauma patients admitted to the intensive care unit (ICU) are particularly vulnerable and prone to increased mortality risk. Thus, accurate prediction of the complications and death probability of trauma patients in ICU could enable early identification and intervention for patients at high mortality risks [3].

Although some prognostic scoring systems such as the Simplified Acute Physiology Score (SAPS) [4] and Acute Physiology and Chronic Health Evaluation (APACHE) [5] exist and are used for risk stratification of ICU patients, and some trauma score instruments such as the Injury Severity Scale (ISS) [6] and Trauma and Injury Severity Score (TRISS) [7] are used for risk stratification of trauma patients, the predictive ability of these scoring systems for mortality trauma patients was still conflicting [8–10]. Besides, two other reasons might hinder the clinical application of these scoring systems. First, these scoring systems' items are too complex, and some of them need to be measured manually. Second, these scoring systems are based on the baseline information (usually admission) to predict an outcome. However, in clinical practice, patient status changes over time, and doctors adjust their prognostic prediction based on the latest status. Hence, a real-time prediction tool based on the latest data outperforms the tools based on baseline data in timeliness and accuracy and actualize precision-medicine-based decision making [11].

Advanced medical equipment can monitor the physiological status of ICU trauma patients in real-time and accumulate massive patient-level temporal data in electronic health record (EHR) systems [12]. In contrast, advanced machine learning techniques are suited to deal with this voluminous data and complex relationships among physiological variables [13]. Bayesian Networks (BNs) have been applied to solve the medical tasks due to their capability to model complex systems in which relationships between the variables were previously completely unknown [14]. Dynamic Bayesian Networks (DBNs) add to BNs the ability to process temporal relationships, and thereby, have become popular in offering an approach to detailed prognostic models that capture the relationships between variables at different time slices and predict the variables in the next time slice from the variables in the previous time slice [15, 16].

Therefore, we sought to develop and validate a real-time prediction model for physiological changes, organ dysfunctions and mortality risk in ICU trauma patients using DBNs based on the massive patient-level temporal data from two centers.

## Materials and methods

### Data collection

Our prediction model was developed in Medical Information Mart for Intensive Care database (MIMIC-III), an ICU database from the Beth Israel Deaconess Medical Center (Boston, MA) [17], and validated using patients at the Burn and Trauma ICU of the Changhai Hospital (ICU-CH) from January 2008 to December 2019, which is one of the major burn and trauma centers in East China [18]. Patients aged > 18 years who received trauma services in ICU were eligible for the study and included in our analysis. Trauma was defined as the injury caused by physical harm from an external source like traffic accident, fight, fall from height and so on. Our study was performed in accordance with the Declaration of Helsinki and the protocol was approved by the Ethics Committee of the Naval Medical University. The MIMIC-III is public de-identifed databases thus informed consent and approval of the Institutional Review Board was waived. Written informed consent was obtained from individual at ICU-CH.

Clinical data were obtained from the patients' electronic health records (EHR). Baseline patients' data at the time of ICU admission were extracted, including age, sex, ICU admission time, ICU discharge time, and in-hospital death time. The physiological items in SAPS II [19] and APACHE II [20] were used as temporal physiological variables in our study. They included temperature, respiratory rate, heart rate, systolic pressure, diastolic pressure, Glasgow coma scale, leukocyte count, platelet count, hematocrit, bilirubin, blood glucose, serum sodium, serum potassium, arterial pH value, serum creatinine, serum urea nitrogen levels, central venous pressure and PO_2_/FIO_2_ Ratio.

### Data preparation

**Fig. 1:**
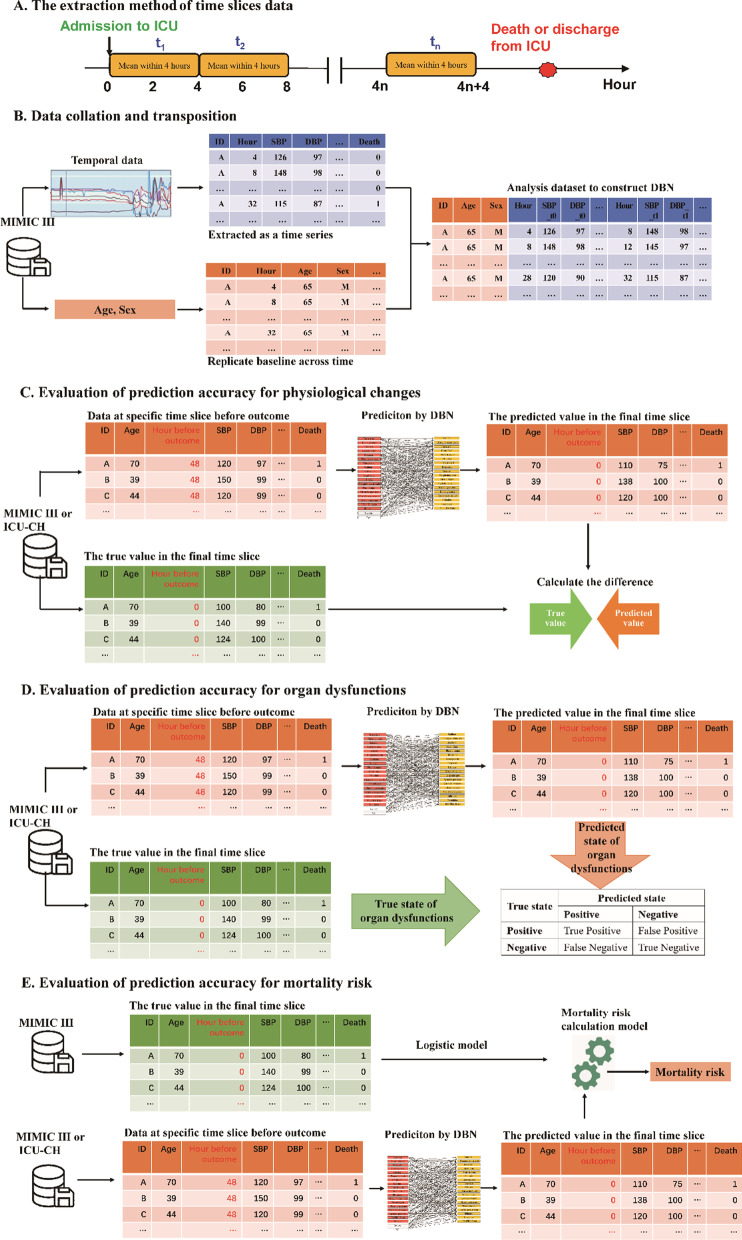
The overview of data extraction, data collation and model evaluation

(1) Outliers’ processing: We considered the influence of outliers in the model construction by setting a series of criteria based on clinical experience to filter out and delete the outliers in the database (Additional file 1: Table S1). (2) The length of time slice: The recording interval of vital signs data range from 15 min to 4 h, and the interval of laboratory tests was about 1 day. The length of the time slice in our DBNs was set to 4 h. If vital signs were measured multiple times in a one-time slice, the average value was used to avoid a fluctuation due to random errors (Fig. 1A). (3) Data collation: All temporal records were organized into longitudinal data by patient identification and time points; baseline data were replicated at each timepoint (Fig. 1B). (4) Normal transformation: Continuous variables should obey normal distribution in DBNs, so all variables were converted to logarithmic values. (5) Data imputation: Different variables were measured with different frequencies, which resulted in missing values of variables at some time points. The missing proportion of temporal physiological variables were shown in Additional file 1: Table S2. For these missing values, the common filling strategy was to impute with the last observation of that variable until the next measurement of the particular value was available or until the end of the time series, and we used this filling strategy in our study [21]. The remaining missing values were imputed with the expectation–maximization algorithm.

### Model development

The construction of a DBN consists of two steps: structure learning and parameter learning. In our study, structure learning of the network was data-driven with some logical constraints. We assumed that the state of physiological variables at time slice *t*1 was only related to the state of the variables at previous time slice *t*0. The DBN structure learning was performed using the PC algorithm, a prototypical constraint-based structure learning method. After building the network structure, we conducted parameter learning to estimate the conditional probabilities that quantify the arcs of the network. Maximum likelihood parameter estimation was used to fit the parameters of DBN.

Our prediction model was built on the collated data derived from MIMIC-III using the DBN. The DBN was implemented using the R package bnlearn. In our study, a simple description of DBN and R codes are shown in the Additional file 1. A detailed description of DBN theory, structure, and parameter learning is provided in a book by Nagarajan et al. [14].

### Model evaluation

The evaluation of our prediction model included three parts: prediction ability of physiological changes, organ dysfunctions and mortality risk at the next 24 h and 48 h.

The DBN model evaluation was conducted in MIMIC-III and ICU-CH, respectively. For patients in ICU stay of > 24 h, we extracted true data in the final time slice (within 4 h before death or discharge) and the last 7th slice (24 h before death or discharge); then we computed the predicted data in a final time slice by DBN after six iteration imputations on data in the last 7th slice. For patients with ICU stay of > 48 h, we extracted real data in the final time slice and the last 13th slice (48 h before death or discharge), and the predicted data in the final time slice were computed by a DBN after 12 iteration imputations on data in the last 13th slice. Then prediction ability of physiological changes, organ dysfunctions and mortality risk at the next 24 h and 48 h was tested in patients' ICU stay for > 24 h and > 48 h, respectively.

For evaluating the prediction accuracy of physiological changes (Fig. 1C), we used the absolute difference and different ratios to measure the distinction between the true and predicted data, which reflected the prediction accuracy of physiological changes for our DBN. For the evaluation of organ dysfunctions risk prediction accuracy (Fig. 1D), the true state of organ dysfunctions was judged by the true physiological data, and the predicted state of organ dysfunctions was judged by the predicted physiological data. The criteria of organ dysfunctions were developed according to Multiple Organ Dysfunction Score (Additional file 1: Table S3). The prediction performance for organ dysfunctions was evaluated by sensitivity, specificity and accuracy. For the evaluation of mortality risk prediction accuracy (Fig. 1E), we used real data in the final time slice of MIMIC-III to build a mortality discrimination model by logistic regression with a restricted cubic spline function. Subsequently, this mortality discrimination model was used to calculate the predicted mortality risk based on the predicted data of the final time slice computed by the DBN. The prediction performance for mortality was evaluated by the areas under the curves (AUCs) of the receiver operating characteristic (ROC) and calibration curves. Moreover, we calculated mortality prediction performance using SAPS II and APACHE II based on the data at the last 7th or 13th slices to compare the prediction ability of mortality between our DBN model and SAPS II and APACHE II scores. The mortality discrimination model, ROC curves, and calibration curves were implemented using R package rms.

In practice, to display intuitively and facilitate the use of our DBN model, an interactive web-based calculator was developed using the R "Shiny" package (https://www.shinyapps.io/).

## Results

In total, we included 2915 ICU admissions from MIMIC- III and 1909 ICU admissions from ICU-CH in this study. The general characteristics of the study participants are shown in Table 1. Cause of injury and ISS from ICU-CH are shown in Additional file 1: Table S4. The structure of our DBN model is shown in Additional file 1: Fig. S1, where the arrows represent the impact path from variables in *t*0 to variables in *t*1.

**Table 1:** Baseline characteristics of study population from MIMIC-III and ICU-CH

Variable	Training set	Validation set
N	2915	1909
Sex, male (%)	1960 (67.2)	995 (52.1)
Death during admission, yes (%)	286 (9.8)	199 (10.4)
Death time during admission		
< 24 h (%)	58 (20.3)	43 (21.6)
24–48 h (%)	42 (14.7)	35 (17.6)
≥ 48 h (%)	186 (65.0)	121 (60.8)
Length of ICU stay, hour		
Median (Q1,Q3)	51.70 (28.95, 121.14)	46.08 (25.87, 98.22)
< 24 h (%)	485 (16.6)	366 (19.2)
24–48 h (%)	893 (30.6)	628 (32.9)
≥ 48 h (%)	1537 (52.7)	915 (47.9)
Primary diagnosis		
Fracture of skull (%)	571 (19.6)	353 (18.5)
Fracture of neck and trunk (%)	574 (19.7)	442 (23.2)
Fracture of limb (%)	310 (10.6)	259 (13.6)
Intracranial injury (%)	570 (19.6)	340 (17.8)
Internal injury of trunk (%)	460 (15.8)	355 (18.6)
Other (%)^a^	428 (14.7)	160 (8.4)
Age, mean ± SD	51.06 ± 22.38	54.54 ± 17.38
SAP II score, mean ± SD	35.15 ± 12.21	34.17 ± 11.74
APACHE II score, mean ± SD	8.63 ± 5.00	6.85 ± 4.48
GCS	10.90 ± 4.26	12.17 ± 2.85

^a^Other included dislocation, sprains and strains of joints and adjacent muscles, open wound of trunk, open wound of limb, etc.

Table 2 shows the prediction accuracy of our DBN model for physiological changes at the 24th hour and 48th hour in MIMIC- III. The difference ratios between the real values and the 24-h predicted values of most physiological variables were within 5%. The errors of 48-h predicted values were slightly larger than that of 24-h predicted values. In the ICU-CH, the different ratios between the real values and the 24-h or 48-h predicted values of all variables were within 15%; indeed, most were within 5% (Table 3). Also, we assessed the prediction accuracy for physiological changes in patients whose outcome was death (Additional file 1: Tables S5, S6). Some physiological variables (like GCS) had large prediction errors in death patients.

**Table 2:** Prediction accuracy of variables at 24th hour and 48th hour in development datasets (MIMIC-III)

Variable name	24th hour				48th hour			
	True value	Predicted value	Difference (95% CI)	Difference ratio (95% CI)^a^	True value	Predicted value	Difference (95% CI)	Difference ratio (95% CI)^a^
Temperature, ℃	37.23 ± 0.72	37.31 ± 0.24	− 0.09 (− 0.12, − 0.06)	− 0.3 (− 0.4, − 0.2)	37.22 ± 0.80	37.36 ± 0.21	− 0.14 (− 0.18, − 0.10)	− 0.4 (− 0.5, − 0.3)
Respiratory rate, beat Per Minute	19.32 ± 4.77	18.93 ± 2.00	0.38 (0.21, 0.55)	− 2.6 (− 3.5, − 1.6)	19.92 ± 5.35	19.40 ± 1.42	0.52 (0.27, 0.77)	− 3.6 (− 4.9, − 2.2)
Heart Rate, beat Per Minute	88.73 ± 15.36	87.39 ± 9.71	1.34 (0.84, 1.84)	− 0.3 (− 0.9, 0.3)	89.25 ± 16.58	87.16 ± 7.36	2.08 (1.38, 2.79)	− 0.3 (− 1.1, 0.6)
Systolic pressure, mmHg	127.49 ± 16.46	127.70 ± 5.61	− 0.21 (− 0.84, 0.42)	− 1.6 (− 2.2, − 1.1)	128.22 ± 17.70	127.55 ± 4.01	0.68 (− 0.21, 1.56)	− 1.3 (− 2.0, − 0.6)
Diastolic pressure, mmHg	63.48 ± 10.84	63.22 ± 5.47	0.26 (− 0.13, 0.65)	− 1.8 (− 2.5, − 1.2)	63.66 ± 11.88	62.62 ± 4.84	1.03 (0.47, 1.59)	− 1.3 (− 2.2, − 0.4)
GCS	11.63 ± 2.98	12.10 ± 2.29	− 0.48 (− 0.58, − 0.38)	− 9.9 (− 11.3, − 8.5)	10.98 ± 3.16	11.55 ± 2.02	− 0.57 (− 0.71, − 0.43)	− 14.3 (− 16.5, − 12.2)
Leukocyte count, K/uL	12.04 ± 4.91	10.97 ± 4.03	1.07 (0.94, 1.21)	4.2 (3.2, 5.3)	12.32 ± 5.01	11.18 ± 3.77	1.13 (0.93, 1.33)	2.3 (0.8, 3.9)
Platelet count, K/uL	263.17 ± 160.94	266.39 ± 171.15	− 3.22 (− 5.69, − 0.75)	− 2.9 (− 3.9, − 1.9)	278.86 ± 179.24	312.20 ± 194.47	− 33.34 (− 38.12, − 28.57)	− 17.8 (− 19.7, − 15.8)
Hematocrit, %	31.26 ± 5.51	30.06 ± 4.16	1.20 (1.06, 1.34)	2.8 (2.4, 3.2)	30.22 ± 4.90	29.21 ± 3.43	1.01 (0.82, 1.20)	2.0 (1.4, 2.7)
Bilirubin, mg/dL	0.95 ± 1.80	0.96 ± 1.65	− 0.01 (− 0.03, 0.01)	− 7.1 (− 9.0, − 5.1)	1.03 ± 1.95	1.01 ± 1.84	0.02 (− 0.01, 0.05)	− 6.1 (− 9.4, − 2.8)
Blood glucose, mg/dL	130.98 ± 37.38	124.59 ± 20.34	6.39 (4.97, 7.81)	1.5 (0.7, 2.3)	133.98 ± 37.85	125.43 ± 15.97	8.55 (6.68, 10.41)	2.6 (1.6, 3.6)
Sodium, mEq/L	139.76 ± 4.20	139.20 ± 3.83	0.56 (0.44, 0.69)	0.4 (0.3, 0.5)	139.82 ± 4.34	139.38 ± 3.73	0.44 (0.25, 0.63)	0.3 (0.1, 0.4)
Potassium, mEq/L	4.00 ± 0.44	3.93 ± 0.28	0.06 (0.04, 0.08)	0.7 (0.3, 1.1)	4.01 ± 0.44	3.94 ± 0.22	0.07 (0.05, 0.09)	0.8 (0.2, 1.3)
PH value	7.40 ± 0.05	7.41 ± 0.03	− 0.01 (− 0.01, − 0.01)	− 0.1 (− 0.1, − 0.1)	7.41 ± 0.06	7.42 ± 0.02	− 0.01 (− 0.01, − 0.01)	− 0.2 (− 0.2, − 0.1)
Creatinine, mg/dL	0.89 ± 1.17	0.81 ± 0.96	0.08 (0.02, 0.13)	2.1 (− 3.9, 8.1)	0.90 ± 1.46	0.79 ± 0.93	0.10 (0.02, 0.19)	2.6 (− 4.7, 9.9)
Urea nitrogen, mg/dL	17.98 ± 12.72	17.03 ± 12.64	0.95 (0.76, 1.13)	3.8 (2.7, 4.9)	19.76 ± 14.42	18.94 ± 13.65	0.82 (0.45, 1.20)	− 1.2 (− 3.2, 0.8)
Central venous pressure, mmHg	8.70 ± 2.11	8.84 ± 1.29	− 0.13 (− 0.20, − 0.06)	− 5.5 (− 7.1, − 3.9)	8.95 ± 2.31	9.10 ± 1.17	− 0.15 (− 0.26, − 0.05)	− 7.5 (− 9.5, − 5.6)
PO_2_/FIO_2_ Ratio, mmHg	283.22 ± 78.87	266.62 ± 43.22	16.61 (13.55, 19.66)	1.3 (0.2, 2.4)	281.52 ± 98.74	257.19 ± 29.49	24.32 (19.62, 29.03)	0.1 (− 1.5, 1.7)

^a^Difference ratio = (True value − Predicted value) × 100%/ True value

**Table 3:** Prediction accuracy of variables at 24th hour and 48th hour in testing dataset (ICU-CH)

Variable name	24th hour				48th hour			
	True value	Predicted value	Difference 95% CI)	Difference ratio (95% CI)^a^	True value	Predicted value	Difference (95% CI)	Difference ratio (95% CI)^a^
Temperature, ℃	36.95 ± 0.65	37.17 ± 0.24	− 0.22 (− 0.25, − 0.18)	− 0.6 (− 0.7, − 0.5)	37.02 ± 0.71	37.25 ± 0.19	− 0.23 (− 0.28, − 0.19)	− 0.7 (− 0.8, − 0.5)
Respiratory rate, beat Per Minute	18.48 ± 4.36	18.52 ± 2.06	− 0.04 (− 0.24, 0.16)	− 3.9 (− 5.0, − 2.8)	18.96 ± 4.40	18.99 ± 1.28	− 0.03 (− 0.30, 0.25)	− 4.7 (− 6.3, − 3.2)
Heart Rate, beat Per Minute	79.31 ± 14.21	82.17 ± 9.49	− 2.86 (− 3.48, − 2.24)	− 5.6 (− 6.4, − 4.7)	80.58 ± 15.30	83.37 ± 6.09	− 2.79 (− 3.69, − 1.90)	− 6.4 (− 7.6, − 5.2)
Systolic pressure, mmHg	127.58 ± 16.64	128.57 ± 6.85	− 0.99 (− 1.82, − 0.16)	− 2.1 (− 2.8, − 1.5)	131.07 ± 18.35	128.43 ± 4.04	2.64 (1.42, 3.86)	0.2 (− 0.7, 1.2)
Diastolic pressure, mmHg	65.22 ± 11.39	63.38 ± 5.51	1.84 (1.31, 2.37)	0.7 (− 0.1, 1.5)	66.50 ± 11.94	62.51 ± 4.10	3.98 (3.24, 4.72)	3.5 (2.4, 4.6)
GCS	12.38 ± 3.04	12.52 ± 2.55	− 0.14 (− 0.25, − 0.02)	− 5.3 (− 6.9, − 3.7)	12.01 ± 3.18	12.03 ± 2.22	− 0.02 (− 0.18, 0.15)	− 6.7 (− 9.1, − 4.4)
Leukocyte count, K/uL	11.09 ± 4.79	10.56 ± 3.95	0.53 (0.37, 0.70)	− 0.3 (− 1.9, 1.2)	11.35 ± 4.77	10.35 ± 3.38	1.00 (0.76, 1.24)	2.0 (− 0.1, 4.0)
Platelet count, K/uL	274.61 ± 125.19	271.43 ± 123.69	3.18 (0.45, 5.92)	− 0.1 (− 1.0, 0.8)	282.15 ± 131.37	294.73 ± 134.14	− 12.57 (− 17.86, − 7.29)	− 7.9 (− 9.8, − 6.0)
Hematocrit, %	33.09 ± 5.58	31.91 ± 4.48	1.18 (1.01, 1.34)	2.8 (2.3, 3.3)	32.57 ± 5.65	30.93 ± 3.90	1.64 (1.39, 1.88)	3.8 (3.1, 4.5)
Bilirubin, mg/dL	0.62 ± 0.90	0.65 ± 0.89	− 0.02 (− 0.04, − 0.01)	− 6.8 (− 8.7, − 4.8)	0.64 ± 0.89	0.67 ± 0.96	− 0.03 (− 0.05, − 0.01)	− 8.2 (− 11.3, − 5.0)
Blood glucose, mg/dL	143.57 ± 58.15	133.68 ± 23.77	9.89 (6.84, 12.95)	2.5 (1.5, 3.6)	139.87 ± 33.67	131.81 ± 18.01	8.06 (6.04, 10.08)	2.7 (1.4, 3.9)
Sodium, mEq/L	138.90 ± 4.28	139.12 ± 4.12	− 0.22 (− 0.41, − 0.03)	− 0.2 (− 0.3, − 0.1)	138.98 ± 4.41	139.02 ± 3.79	− 0.04 (− 0.30, 0.21)	− 0.1 (− 0.3, 0.1)
Potassium, mEq/L	3.96 ± 0.44	3.93 ± 0.30	0.03 (0.01, 0.05)	− 0.1 (− 0.6, 0.5)	3.93 ± 0.45	3.92 ± 0.20	0.01 (− 0.02, 0.04)	− 0.7 (− 1.4, − 0.0)
PH value	7.43 ± 0.04	7.42 ± 0.03	0.00 (0.00, 0.01)	0.1 (0.0, 0.1)	7.44 ± 0.04	7.43 ± 0.02	0.01 (0.01, 0.02)	0.2 (0.1, 0.2)
Creatinine, mg/dL	0.85 ± 0.56	0.81 ± 0.48	0.05 (0.04, 0.06)	3.7 (2.8, 4.6)	0.84 ± 0.53	0.78 ± 0.45	0.07 (0.05, 0.08)	5.1 (3.8, 6.3)
Urea nitrogen, mg/dL	17.50 ± 11.35	17.14 ± 11.29	0.36 (0.13, 0.59)	− 0.7(− 2.0, 0.7)	17.99 ± 12.25	18.00 ± 11.85	− 0.01 (− 0.44, 0.41)	− 5.4 (− 7.7, − 3.2)
Central venous pressure, mmHg	7.83 ± 1.87	8.24 ± 1.17	− 0.41 (− 0.48, − 0.34)	− 9.6 (− 12.1, − 7.1)	7.96 ± 2.30	8.59 ± 1.04	− 0.64 (− 0.77, − 0.51)	− 14.6 (− 16.9, − 12.4)
PO_2_/FIO_2_ Ratio, mmHg	323.16 ± 70.65	289.80 ± 42.39	33.36 (29.79, 36.94)	7.8 (6.7, 8.8)	308.68 ± 73.75	272.85 ± 25.43	35.84 (31.17, 40.50)	7.5 (5.9, 9.2)

^a^Difference ratio = (True value − Predicted value) × 100%/True value

**As** Table 4 shown, our model had good predicting ability for predicting renal, hepatic, cardiovascular and hematologic dysfunctions with accuracy more than 0.8 in MIMIC- III and ICU-CH. For the 48-h neurological dysfunction in MIMIC- III and the respiratory dysfunction in ICU-CH, the prediction accuracy of our DBN model was less than 0.8.

**Table 4:** Prediction accuracy of organ dysfunctions risk at 24th hour and 48th hour

Organ dysfunction	24th hour			48th hour		
	Sensitivity	Specificity	Accuracy	Sensitivity	Specificity	Accuracy
MIMIC-III						
Respiratory	0.920	0.784	0.881	0.951	0.763	0.900
Renal	0.735	0.985	0.953	0.821	0.989	0.967
Hepatic	0.767	0.950	0.922	0.751	0.960	0.924
Cardiovascular	0.724	0.914	0.873	0.676	0.872	0.813
Hematologic	0.742	0.961	0.939	0.631	0.985	0.943
Neurologic	0.719	0.947	0.859	0.679	0.777	0.725
ICU-CH						
Respiratory	0.852	0.739	0.781	0.951	0.634	0.796
Renal	0.715	0.984	0.950	0.784	0.990	0.965
Hepatic	0.707	0.983	0.970	0.690	0.989	0.967
Cardiovascular	0.761	0.961	0.938	0.632	0.946	0.889
Hematologic	0.773	0.991	0.979	0.711	0.993	0.977
Neurologic	0.775	0.955	0.903	0.764	0.858	0.824

Sensitivity = (true positives)/(true positives + false negatives), specificity = (true negatives)/(true negatives + false positives), accuracy = (true positives + true negatives)/total

**Fig. 2:**
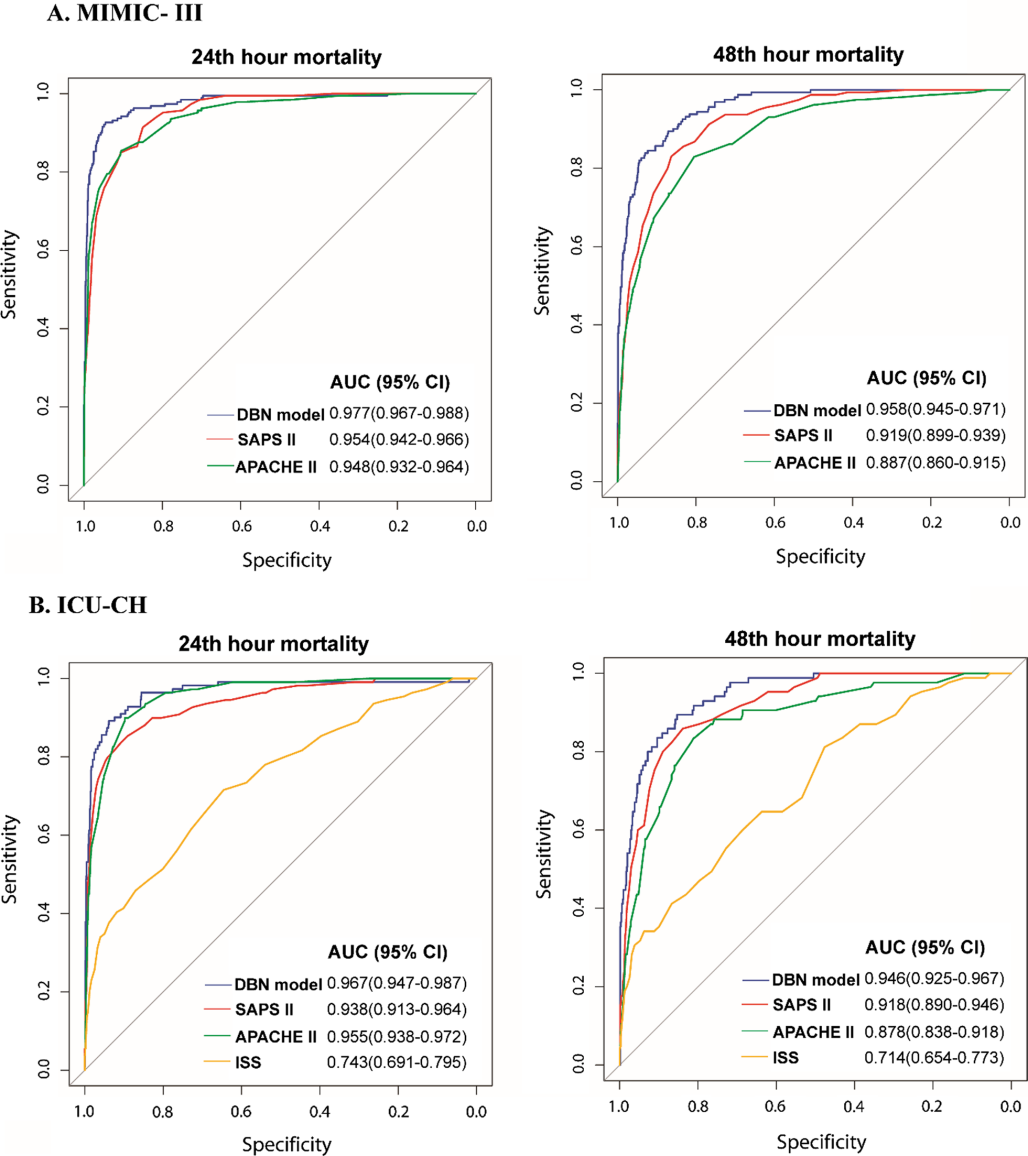
Comparison of prediction models by ROC analysis

Figure 2 shows the prediction accuracy of our DBN model for mortality risk. In MIMIC-III, the AUC of the mortality discrimination model using the data predicted by DBN based on the 24th hour data before outcome was 0.977 (95%CI, 0.967–0.988). The AUCs of SAPS-II and APACHE-II based on the 24th hour data before outcome were 0.954 (95%CI, 0.942–0.966) and 0.948 (95%CI, 0.932–0.964). In a similar scenario, the AUC of the model using data predicted by DBN was higher than that of other models, and appeared in the 48-h mortality prediction in MIMIC- III and 24-h and 48-h mortality prediction in ICU-CH. Calibration plots in Additional file 1: Fig. S2 showed that the predicted mortality from the model using data predicted by DBN closely approximated the actual outcomes.

We developed a web-based calculator based on our DBN model to predict physiological changes and mortality risk for new trauma patients available at the website https://jsong67.shinyapps.io/Prediction2/. This web-based calculator requires the input of the participant's baseline characteristics and physiological variables and then outputs the predicted results.

## Discussion

Our study built a DBN model for predicting physiological changes, organ dysfunctions and mortality risk in critical trauma patients and validated the model in an external dataset with good discrimination and calibration. The DBN model was based on the variables in SAPS-II and APACHE-II and is accessible online by a web application. Compared with other machine learning-based models, our model can be readily calculated with a web application that allows clinicians to use our model in practice and help to validate our model in their medical work.

In practice, a trauma patient's current physiological variables' values could be inputted in our DBN model to calculate and physiological changes, organ dysfunctions and the death risk in the future 24 and 48 h. As more physiological variables become available during ICU monitoring, our DBN model is able to update the predicted values dynamically. With the emergence of personalized medicine, our DBN model can not only predict the risk of death, but also predict physiological variables to predict the occurrence of organ dysfunctions. Then, our model can be used for clinical decision making, with a view of early interventions, thereby preventing a delay in the initiation of appropriate therapy that has been recognized as a risk factor for mortality among ICU patients [22, 23].

The relationship between ICU patients' physiological variables is highly complex (usually nonlinear and interactive), which is unlikely to be captured by common parametric methods (e.g., linear regression). Moreover, models designed to be intuitive for human experts' understanding may not be computationally efficient or accurate for probabilistic modeling [24]. Methods that consider the complex conditional inter-dependencies between variables would be more precise in probabilistic modeling. The DBN extends standard BNs with the concept of time and can handle arbitrary nonlinear and complicated time-dependent relationships, which can be used for a wide range of tasks, including prediction and decision making under uncertainty [25, 26]. Our study demonstrated that the DBN is a robust method, able to predict physiological changes and improve the prediction accuracy of mortality compared with traditional tools like the SAPS-II and APACHE-II.

Compared with other studies that directly put outcome variables into the DBN [15, 16, 25, 26], our study did not use this approach for two reasons. First, there is about 10% of patients' death in our dataset (Table 1). If the mortality was included in the DBN model, it would lead to an imbalance in machine learning, causing overfitting and reducing the external accuracy [27]. Second, deaths are discrete data, while physiological variables are continuous data, and the combination of these data types yield mixed data. A simple way to learn DBN from mixed data is to convert all continuous variables to discrete ones [14]. However, there are many discretization methods; thus, it is difficult to determine the appropriate discretization mode; also, our study's purpose was to predict the specific value of physiological indicators. Therefore, death was not put into the DBN model, and the mortality risk was calculated based on the predicted physiological values.

There are four major limitations to our study. First, we included the variables in SAPS-II and APACHE-II, although more recent versions of SAPS and APACHE are available, partly because some variables in recent versions do not exist in our two databases, and partly because we wanted to keep our model as simple as possible. Second, some complications such as sepsis were not included in our model since the lack of the occurrence time of complications in our datasets. Third, although our DBN model performed well in external validation, our data were from two high-level hospitals with advanced medical conditions and rich medical experiences. The physiological changes are not only affected by trauma but also affected by medical conditions. So, our DBN model still needs extensive validation in various types of hospitals in the future. Finally, participants with the same input values had the same output values calculated using the DBN model, but each person is unique with individual characteristics. Therefore, although our DBN prediction model could support decision making, not all medical care decisions need to be made by a clinician.

## Conclusion

Our DBN model can be used as a real-time prediction tool to predict physiological changes, organ dysfunctions and mortality risk in ICU trauma patients and achieve better performance than conventional severity scores. Moreover, our study demonstrates that the DBN is a promising method for predicting of medical temporal data. In the future, we should validate our DBN model to verify its prediction accuracy and further improve the web calculator to increase the user convenience of the models by a physician.

Supplement tables show the definition of non-fault value, the missing proportion of variables in MIMIC-III, the criteria of organ dysfunctions, cause of injury and Injury Severity Score from ICU-CH, and prediction accuracy of physiological variables at 24th hour and 48th hour in death population. Supplement figures show the structure of DBN model and calibration curves. Supplement texts show R codes of dynamic Bayesian network.

## Supplementary Information

**Additional file 1: Table S1**. The definition of non-fault value. **Table S2**. The missing proportion of temporal physiological variables in MIMIC-III. **Table S3**. The criteria of organ dysfunctions. **Table S4**. Cause of injury and Injury Severity Score from ICU-CH. **Table S5**. Prediction accuracy of variables at 24th hour and 48th hour in death population from development datasets (MIMICIII). **Table S6**. Prediction accuracy of variables at 24th hour and 48th hour in death population from testing dataset (ICU-CH). **Figure S1**. Prediction accuracy of variables at 24th hour and 48th hour in death population from testing dataset (ICU-CH). **Figure S2**. Calibration curves. **Supplement texts**. The description and R codes of dynamic Bayesian network.

## Data Availability

The MIMIC-III analyzed during our study are available in the PhysioNet repository, [https://mimic.mit.edu/]. The ICU-CH are not publicly available due to privacy and ethical restrictions but are available from the corresponding author on reasonable request.
