# Supplementary material for: Dynamic Bayesian network for predicting physiological changes, organ dysfunctions and mortality risk in critical trauma patients
Source: BMC Med Inform Decis Mak. 2022 May 3;22:119. doi: 10.1186/s12911-022-01803-y (PMC9063308; doi:10.1186/s12911-022-01803-y)
Supplement: Supplementary file 1 — Additional file 1: Table S1. The definition of non-fault value. Table S2. The missing proportion of temporal physiological variables in MIMIC-III. Table S3. The criteria of organ dysfunctions. Table S4. Cause of injury and Injury Severity Score from ICU-CH. Table S5. Prediction accuracy of variables at 24th hour and 48th hour in death population from development datasets (MIMICIII). Table S6. Prediction accuracy of variables at 24th hour and 48th hour in death population from testing dataset (ICU-CH). Figure S1. Prediction accuracy of variables at 24th hour and 48th hour in death population from testing dataset (ICU-CH). Figure S2. Calibration curves. Supplement texts. The description and R codes of dynamic Bayesian network. [file 12911_2022_1803_MOESM1_ESM.docx]

**Supplement tables**

Supplement table 1. The definition of non-fault value

| Index | Unit | Lower limit | Upper limit |
| --- | --- | --- | --- |
| Temperature | ℃ | 25 | 44 |
| Respiratory rate | Beat Per Minute | 0 | 50 |
| Heart rate | Beat Per Minute | 0 | 200 |
| Systolic pressure | mmHg | 0 | 250 |
| Diastolic pressure | mmHg | 0 | 250 |
| Glasgow Coma Scale | - | 3 | 15 |
| Leukocyte count | K/uL | 1 | 80 |
| Platelet count | K/uL | 10 | 1300 |
| Hematocrit | % | 10 | 90 |
| Bilirubin | mg/dL | 0.1 | 20 |
| Blood glucose | mg/dL | 10 | 1000 |
| Sodium | mEq/L | 100 | 200 |
| Potassium | mEq/L | 1 | 30 |
| PH value | - | 4 | 10 |
| Creatinine | mg/dL | 0.2 | 60 |
| Urea nitrogen | mg/dL | 2 | 130 |
| Central venous pressure | mmHg | 0 | 30 |
| PO_2_/FIO_2_ Ratio | - | 0 | 1000 |

Supplement table 2. The missing proportion of temporal physiological variables in MIMIC-III

| Index | Missing proportion |
| --- | --- |
| Temperature | 10.17% |
| Respiratory rate | 5.15% |
| Heart rate | 4.89% |
| Systolic pressure | 5.52% |
| Diastolic pressure | 5.52% |
| Glasgow Coma Scale | 10.49% |
| Leukocyte count | 6.25% |
| Platelet count | 6.24% |
| Hematocrit | 6.68% |
| Bilirubin | 63.92% |
| Blood glucose | 4.84% |
| Sodium | 5.61% |
| Potassium | 5.06% |
| PH value | 33.42% |
| Creatinine | 5.83% |
| Urea nitrogen | 5.92% |
| PO_2_/FIO_2_ Ratio | 61.24% |
| Central venous pressure | 57.24% |

The missing proportion were calculated based on the data from the ICU admission to death or ICU discharge

Supplement table 3. The criteria of organ dysfunctions

| Organ system | Diagnostic variables | Criteria |
| --- | --- | --- |
| Respiratory | PO_2_/FIO_2_ Ratio | ≤300 |
| Renal | Creatinine | >100 |
| Hepatic | Bilirubin | >20 |
| Cardiovascular | R/P Ratio | >10 |
| Hematologic | Platelet count | ≤120 |
| Neurologic | Glasgow Coma Score | <15 |

The R/P ratio is calculated as the product of the heart rate and central venous pressure, divided by the mean arterial pressure. The criteria of organ system score ≥ 1 in Multiple Organ Dysfunction Score system was used as the diagnostic criteria of organ failure

Supplement table 4. Cause of injury and Injury Severity Score from ICU-CH

| Variable | N | Percent |
| --- | --- | --- |
| Cause of injury |  |  |
| Traffic accident | 1057 | 55.4 |
| Fight | 327 | 17.1 |
| Fall from height | 189 | 9.9 |
| Slip and fall | 121 | 6.3 |
| Other* | 215 | 11.3 |
| Injury Severity Score, ISS |  |  |
| <16 | 201 | 10.5 |
| 16-25 | 672 | 35.2 |
| 25-35 | 833 | 43.6 |
| 35-50 | 199 | 10.4 |
| ≥50 | 4 | 0.2 |

*Other included burn, electric shock, suicide, industrial trauma, etc.

Supplement table 5. Prediction accuracy of variables at 24th hour and 48th hour in death population from development datasets (MIMIC-III)

|  | 24th hour | | | | 48th hour | | | |
| --- | --- | --- | --- | --- | --- | --- | --- | --- |
| Variable name | True value | Predicted value | Difference  (95% CI) | Difference ratio  (95% CI)* | True value | Predicted value | Difference  (95% CI) | Difference ratio  (95% CI)* |
| Temperature, ℃ | 37.22±0.97 | 37.44±0.32 | -0.22(-0.35,-0.09) | -0.6(-1.0,-0.3) | 37.28±1.00 | 37.44±0.25 | -0.16(-0.32,-0.01) | -0.5(-0.9,-0.1) |
| Respiratory rate, beat Per Minute | 21.00±5.72 | 20.04±2.65 | 0.96(0.35,1.57) | -0.1(-3.2,3.0) | 21.79±6.24 | 20.11±1.57 | 1.68(0.82,2.53) | 1.4(-2.3,5.1) |
| Heart Rate, beat Per Minute | 86.62±16.71 | 87.75±12.30 | -1.13(-3.02,0.77) | -3.4(-5.7,-1.2) | 86.31±16.79 | 86.60±8.26 | -0.28(-2.59,2.02) | -3.6(-6.6,-0.5) |
| Systolic pressure, mmHg | 126.56±18.41 | 124.87±8.82 | 1.69(-0.50,3.89) | -0.2(-2.0,1.6) | 128.04±19.67 | 125.42±5.85 | 2.62(-0.16,5.40) | 0.0(-2.2,2.2) |
| Diastolic pressure, mmHg | 60.83±10.49 | 59.54±6.71 | 1.29(0.15,2.43) | 0.4(-1.5,2.3) | 60.46±11.01 | 58.94±5.33 | 1.52(0.10,2.95) | 0.1(-2.4,2.5) |
| GCS | 6.77±3.36 | 7.02±2.45 | -0.24(-0.59,0.10) | -18.7(-24.7,-12.8) | 6.72±3.27 | 7.76±1.95 | -1.04(-1.44,-0.65) | -36.6(-45.1,-28.1) |
| Leukocyte count, K/uL | 13.94±6.90 | 13.75±6.69 | 0.18(-0.39,0.76) | -2.8(-6.9,1.4) | 13.59±5.65 | 13.11±5.42 | 0.48(-0.21,1.17) | -2.6(-8.0,2.9) |
| Platelet count, K/uL | 212.65±117.42 | 209.25±128.04 | 3.40(-4.25,11.05) | -0.5(-4.3,3.4) | 222.97±131.48 | 231.49±144.86 | -8.52(-20.59,3.56) | -8.3(-13.7,-3.0) |
| Hematocrit, % | 30.57±5.34 | 29.39±3.92 | 1.18(0.75,1.61) | 2.9(1.6,4.2) | 30.63±5.33 | 28.78±3.27 | 1.84(1.25,2.43) | 4.7(3.0,6.4) |
| Bilirubin, mg/dL | 1.87±5.73 | 1.83±5.35 | 0.04(-0.06,0.15) | -7.9(-14.7,-1.0) | 1.99±5.36 | 2.04±5.27 | -0.06(-0.24,0.13) | -14.0(-22.9,-5.2) |
| Blood glucose, mg/dL | 142.92±38.52 | 137.87±25.00 | 5.05(-0.47,10.58) | -0.2(-3.3,2.9) | 149.30±48.81 | 135.05±19.46 | 14.24(6.64,21.85) | 4.2(0.7,7.7) |
| Sodium, mEq/L | 142.72±7.18 | 143.33±6.83 | -0.61(-1.30,0.08) | -0.5(-1.0,-0.0) | 141.92±6.38 | 143.05±5.95 | -1.13(-2.07,-0.19) | -0.9(-1.5,-0.2) |
| Potassium, mEq/L | 4.08±0.56 | 4.03±0.41 | 0.05(-0.01,0.12) | 0.4(-1.2,2.0) | 4.07±0.55 | 4.01±0.30 | 0.06(-0.01,0.13) | 0.3(-1.3,2.0) |
| PH value | 7.39±0.07 | 7.40±0.06 | -0.01(-0.02,-0.00) | -0.1(-0.3,-0.0) | 7.40±0.08 | 7.41±0.05 | -0.01(-0.02,-0.00) | -0.2(-0.3,-0.0) |
| Creatinine, mg/dL | 1.21±1.07 | 1.41±3.12 | -0.20(-0.63,0.24) | -39.0(-111.4,33.4) | 1.21±1.04 | 1.37±2.69 | -0.15(-0.57,0.26) | -35.4(-103.2,32.3) |
| Urea nitrogen, mg/dL | 29.04±20.64 | 30.45±21.09 | -1.40(-2.28,-0.53) | -7.9(-11.8,-4.0) | 30.00±21.78 | 32.81±20.84 | -2.80(-4.57,-1.04) | -21.2(-28.4,-14.0) |
| Central venous pressure, mmHg | 10.94±3.74 | 10.46±2.31 | 0.48(0.07,0.88) | -1.8(-6.5,2.9) | 10.54±3.67 | 10.45±1.67 | 0.09(-0.37,0.56) | -10.0(-17.6,-2.4) |
| PO_2_/FIO_2_ Ratio, mmHg | 302.08±131.62 | 279.01±66.33 | 23.07(7.25,38.88) | -3.9(-10.0,2.3) | 292.26±114.65 | 259.76±38.70 | 32.50(16.26,48.74) | 0.1(-5.3,5.5) |

*Difference ratio= (True value- Predicted value) ×100%/ True value

Supplement table 6. Prediction accuracy of variables at 24th hour and 48th hour in death population from testing dataset (ICU-CH)

|  | 24th hour | | | | 48th hour | | | |
| --- | --- | --- | --- | --- | --- | --- | --- | --- |
| Variable name | True value | Predicted value | Difference  (95% CI) | Difference ratio  (95% CI)* | True value | Predicted value | Difference  (95% CI) | Difference ratio  (95% CI)* |
| Temperature, ℃ | 37.30±0.73 | 37.41±0.26 | -0.11(-0.23,0.01) | -0.3(-0.7,0.0) | 37.09±0.98 | 37.38±0.23 | -0.29(-0.50,-0.09) | -0.9(-1.4,-0.3) |
| Respiratory rate, beat Per Minute | 20.62±5.27 | 19.68±2.18 | 0.94(0.12,1.76) | 0.6(-2.8,4.0) | 20.62±5.93 | 19.81±1.43 | 0.81(-0.38,2.00) | -3.2(-9.2,2.7) |
| Heart Rate, beat Per Minute | 83.54±13.76 | 85.14±9.11 | -1.60(-3.80,0.60) | -3.7(-6.5,-0.8) | 83.68±18.21 | 85.14±6.30 | -1.46(-4.90,1.97) | -5.4(-9.5,-1.2) |
| Systolic pressure, mmHg | 129.18±17.78 | 126.22±7.75 | 2.96(-0.14,6.06) | 0.7(-1.8,3.2) | 132.43±23.35 | 126.58±5.04 | 5.85(1.27,10.43) | 1.7(-1.9,5.2) |
| Diastolic pressure, mmHg | 61.66±10.62 | 59.41±5.11 | 2.25(0.57,3.94) | 1.6(-1.1,4.3) | 63.33±14.24 | 58.76±4.13 | 4.57(1.82,7.31) | 3.6(-0.3,7.5) |
| GCS | 7.67±3.61 | 7.38±2.56 | 0.29(-0.26,0.84) | -10.2(-17.8,-2.6) | 7.60±3.46 | 8.34±2.26 | -0.74(-1.43,-0.06) | -28.0(-39.0,-16.9) |
| Leukocyte count, K/uL | 13.60±6.38 | 13.22±5.73 | 0.38(-0.30,1.06) | -1.4(-7.0,4.3) | 12.83±6.48 | 12.59±4.79 | 0.24(-0.47,0.96) | -3.6(-8.9,1.7) |
| Platelet count, K/uL | 237.05±131.24 | 232.11±129.31 | 4.94(-2.85,12.74) | 0.7(-2.6,3.9) | 243.99±130.94 | 247.24±135.52 | -3.25(-18.17,11.67) | -4.5(-10.1,1.1) |
| Hematocrit, % | 31.42±5.88 | 29.90±4.53 | 1.53(0.98,2.08) | 4.0(2.5,5.5) | 31.21±5.94 | 28.99±3.89 | 2.21(1.46,2.96) | 5.7(3.5,8.0) |
| Bilirubin, mg/dL | 1.20±2.78 | 1.28±2.68 | -0.09(-0.21,0.04) | -13.9(-24.4,-3.5) | 1.25±2.51 | 1.46±2.68 | -0.21(-0.41,-0.02) | -31.4(-53.2,-9.5) |
| Blood glucose, mg/dL | 177.62±120.71 | 146.65±29.36 | 30.97(8.36,53.58) | 7.9(4.1,11.7) | 155.02±31.59 | 144.82±22.96 | 10.19(3.63,16.76) | 3.8(-0.6,8.2) |
| Sodium, mEq/L | 141.39±6.15 | 142.74±7.48 | -1.35(-2.49,-0.22) | -1.0(-1.8,-0.2) | 141.28±5.88 | 142.08±6.37 | -0.79(-2.11,0.53) | -0.6(-1.6,0.3) |
| Potassium, mEq/L | 3.98±0.45 | 3.97±0.33 | 0.01(-0.06,0.07) | -0.5(-2.3,1.3) | 4.11±0.44 | 3.96±0.25 | 0.15(0.07,0.23) | 2.9(0.9,4.8) |
| PH value | 7.42±0.06 | 7.41±0.06 | 0.01(-0.00,0.02) | 0.1(-0.0,0.3) | 7.43±0.06 | 7.42±0.04 | 0.01(-0.00,0.02) | 0.1(-0.1,0.2) |
| Creatinine, mg/dL | 1.10±0.82 | 1.13±0.83 | -0.02(-0.06,0.02) | -2.7(-6.2,0.9) | 1.05±0.76 | 1.07±0.76 | -0.02(-0.08,0.03) | -4.0(-8.8,0.9) |
| Urea nitrogen, mg/dL | 26.16±18.31 | 27.35±18.10 | -1.18(-1.88,-0.49) | -7.0(-10.3,-3.8) | 25.87±17.82 | 29.04±17.87 | -3.17(-4.37,-1.96) | -19.2(-25.8,-12.5) |
| Central venous pressure, mmHg | 9.68±3.18 | 9.71±1.50 | -0.03(-0.50,0.44) | -7.2(-12.2,-2.2) | 10.00±3.38 | 9.86±1.32 | 0.14(-0.49,0.78) | -7.1(-14.0,-0.2) |
| PO_2_/FIO_2_ Ratio, mmHg | 307.38±85.93 | 289.45±57.30 | 17.94(1.95,33.93) | -0.5(-7.3,6.2) | 309.94±97.64 | 272.23±33.48 | 37.71(17.27,58.15) | 1.9(-7.4,11.1) |

*Difference ratio= (True value- Predicted value) ×100%/ True value

**Supplement figures**


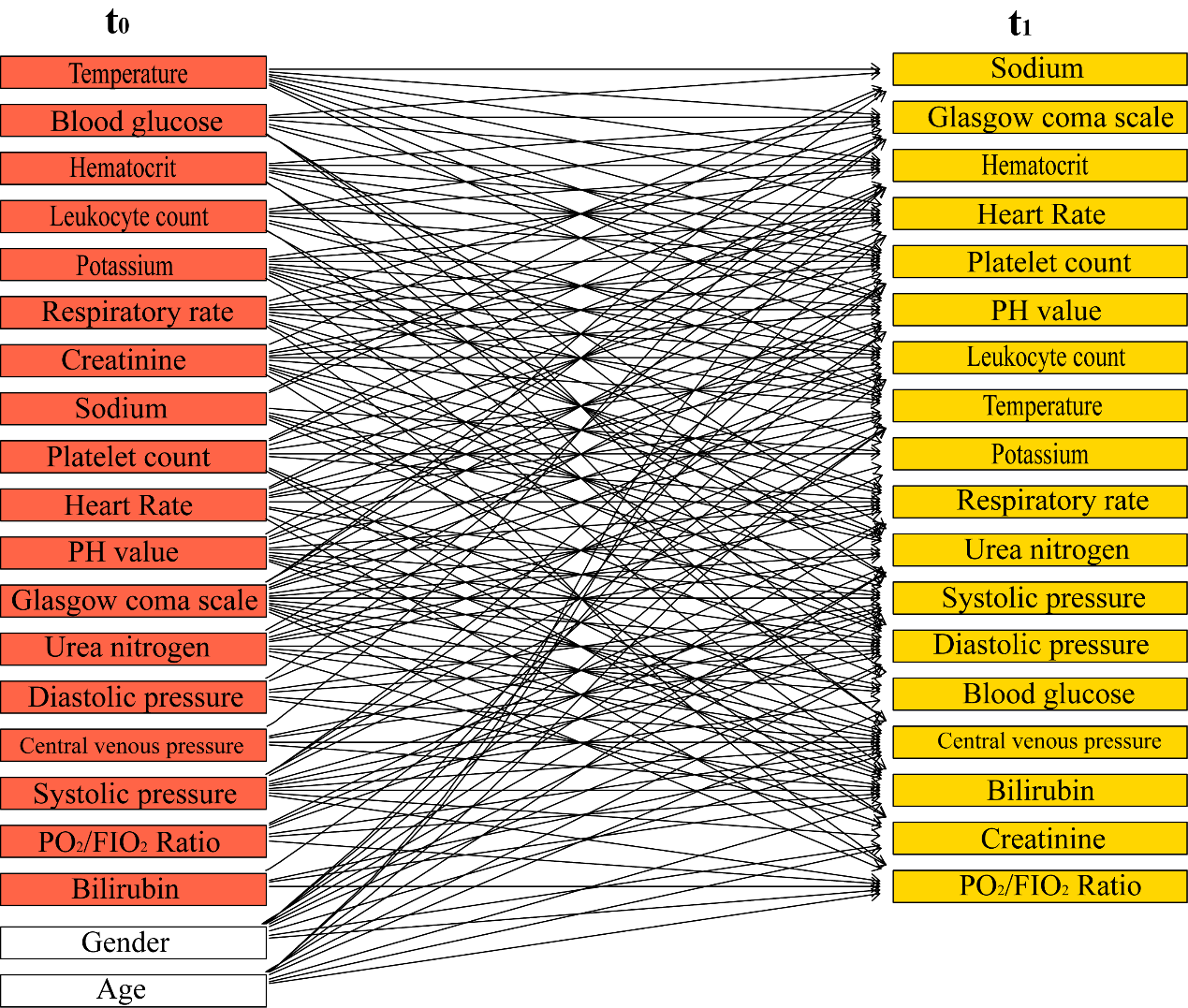


Supplement Figure1. The structure of our dynamic Bayesian network model. The state of physiological variables at time t1 (yellow background) were only related to the state of the variables at t0 (red background) and constant variables (white background).


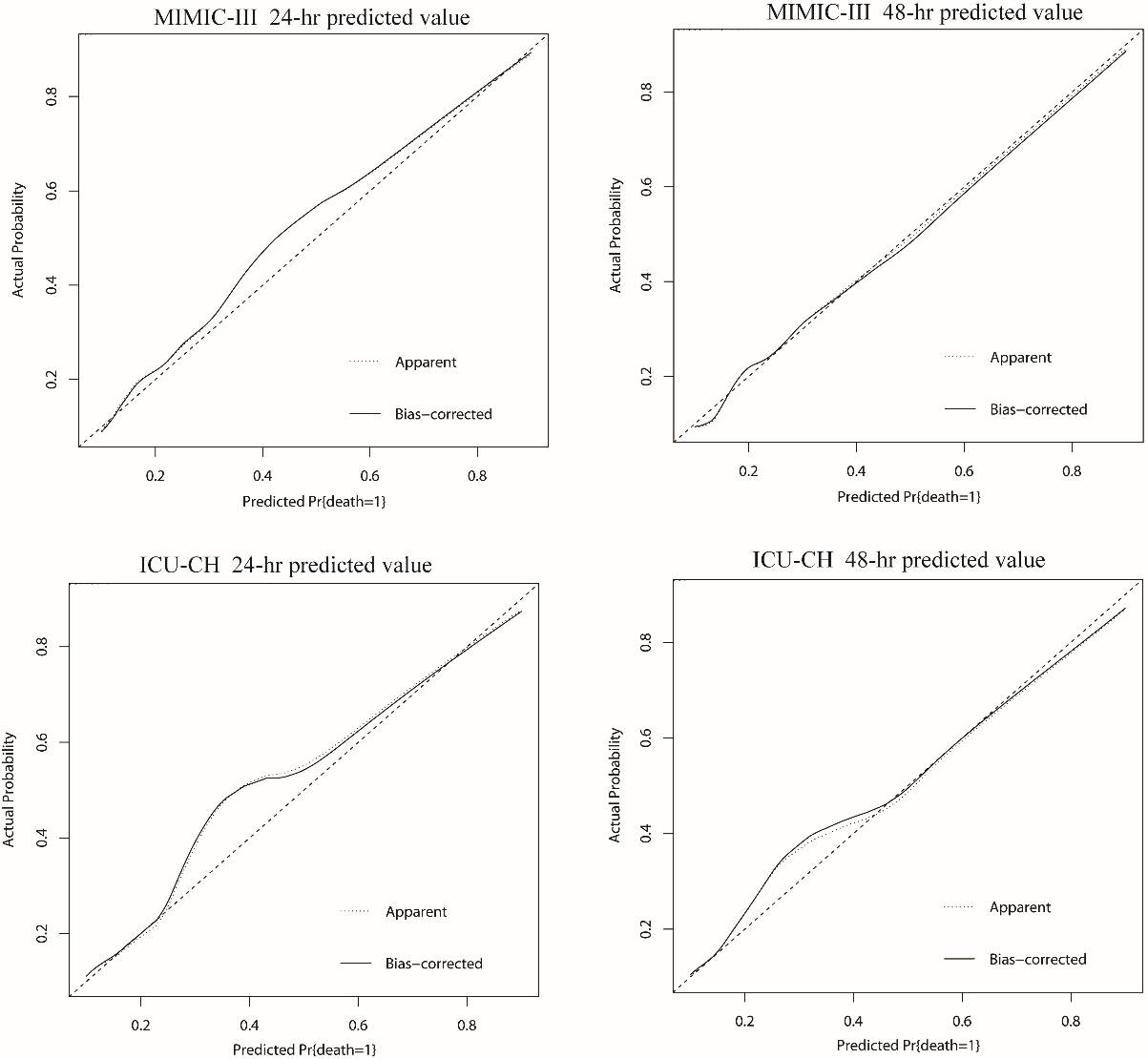


Supplement Figure 2. Calibration curves

**Supplement texts**

**A simple description of dynamic Bayesian network**

A DBN is an extension of the Bayesian network, displaying complicated relationships of variables across time slices. A DBN consists of a set of nodes and a set of arcs; each node represents a variable, whereas arcs represent conditional probabilistic relationships among variables. DBN structure is assumed to be time-invariant and identical across time slices, implying that an influence path between variables does not change over time. Furthermore, a DBN follows the Markov property, in that the state of each variable at time slice *t*1 depends only on the parents at the previous time slice *t*0 but not earlier time slices.

**The** **R codes** **of** **dynamic Bayesian network**

######################### DBN construction#######################

# Load package and data

library(bnlearn)

dbn_dis<-read.table("E:/analysisdataset.csv",header=TRUE,sep=",")

# Set up variable type

# including constant variables, temporal variables at t0 and temporal variables at t1

const <- c("var21_t0","var22_t0") # age and sex

t0 <- c( "var1_t0", "var2_t0", "var3_t0", "var4_t0", "var5_t0",

"var6_t0", "var8_t0","var9_t0", "var10_t0", "var12_t0",

"var13_t0", "var14_t0","var15_t0", "var16_t0", "var17_t0",

"var18_t0", "var26_t0", "var27_t0")# physiological variables at time t0

t1 <- c( "var1_t1", "var2_t1", "var3_t1", "var4_t1", "var5_t1",

"var6_t1", "var8_t1","var9_t1", "var10_t1", "var12_t1",

"var13_t1", "var14_t1","var15_t1", "var16_t1", "var17_t1",

"var18_t1", "var26_t1", "var27_t1")# physiological variables at time t1

# Set up whitelist and blacklist

empty.t1 = expand.grid(from = c(t1,const, t0), to = c(const, t0), stringsAsFactors = FALSE)

t1tot1 =expand.grid(from = t1, to = t1, stringsAsFactors = FALSE)

bl = rbind(tiers2blacklist(list(t0, t1)), empty.t1,t1tot1)

wl <- data.frame(from =t0, to = t1)

# structure learning using PC algorithm

dbn.pc<-pc.stable(dbn_dis, whitelist = wl, blacklist = bl)

# parameter learning

pc.fit<-bn.fit(dbn.pc,dbn_dis)

######################obtain the predicted values by DBN######################

#Import data at a specific time point (t0) and predict the values after 24 hours (t6)

dbn_dis24<-read.table("E:/currentvalue.csv",header=TRUE,sep=",")

#Build dataset with variables at t0 and variables at t1 (no data)

dbn_dis24_t1_1<-rep(NA, N) #N is the number of people in the data

<-data.frame(var1_t1=as.numeric(dbn_dis24_t1_1),

var2_t1=as.numeric(dbn_dis24_t1_1),

var3_t1=as.numeric(dbn_dis24_t1_1),

var4_t1=as.numeric(dbn_dis24_t1_1),

var5_t1=as.numeric(dbn_dis24_t1_1),

var6_t1=as.numeric(dbn_dis24_t1_1),

var8_t1=as.numeric(dbn_dis24_t1_1),

var9_t1=as.numeric(dbn_dis24_t1_1),

var10_t1=as.numeric(dbn_dis24_t1_1),

var12_t1=as.numeric(dbn_dis24_t1_1),

var13_t1=as.numeric(dbn_dis24_t1_1),

var14_t1=as.numeric(dbn_dis24_t1_1),

var15_t1=as.numeric(dbn_dis24_t1_1),

var16_t1=as.numeric(dbn_dis24_t1_1),

var17_t1=as.numeric(dbn_dis24_t1_1),

var18_t1=as.numeric(dbn_dis24_t1_1),

var26_t1=as.numeric(dbn_dis24_t1_1),

var27_t1=as.numeric(dbn_dis24_t1_1))

dbn_dis24<-cbind(dbn_dis24,dbn_dis24_t1)

# Iterative prediction, one iteration represents 4-hour prediction

dbn.impute<-list();

dbn.impute[[1]]<-impute(pc.fit,dbn_dis24)

for (i in 1:5)

{

dbn.impute[[i+1]]<-dbn.impute[[i]];

dbn.impute[[i+1]]$var1_t0<-dbn.impute[[i]]$var1_t1;

dbn.impute[[i+1]]$var2_t0<-dbn.impute[[i]]$var2_t1;

dbn.impute[[i+1]]$var3_t0<-dbn.impute[[i]]$var3_t1;

dbn.impute[[i+1]]$var4_t0<-dbn.impute[[i]]$var4_t1;

dbn.impute[[i+1]]$var5_t0<-dbn.impute[[i]]$var5_t1;

dbn.impute[[i+1]]$var6_t0<-dbn.impute[[i]]$var6_t1;

dbn.impute[[i+1]]$var8_t0<-dbn.impute[[i]]$var8_t1;

dbn.impute[[i+1]]$var9_t0<-dbn.impute[[i]]$var9_t1;

dbn.impute[[i+1]]$var10_t0<-dbn.impute[[i]]$var10_t1;

dbn.impute[[i+1]]$var12_t0<-dbn.impute[[i]]$var12_t1;

dbn.impute[[i+1]]$var13_t0<-dbn.impute[[i]]$var13_t1;

dbn.impute[[i+1]]$var14_t0<-dbn.impute[[i]]$var14_t1;

dbn.impute[[i+1]]$var15_t0<-dbn.impute[[i]]$var15_t1;

dbn.impute[[i+1]]$var16_t0<-dbn.impute[[i]]$var16_t1;

dbn.impute[[i+1]]$var17_t0<-dbn.impute[[i]]$var17_t1;

dbn.impute[[i+1]]$var18_t0<-dbn.impute[[i]]$var18_t1;

dbn.impute[[i+1]]$var26_t0<-dbn.impute[[i]]$var26_t1;

dbn.impute[[i+1]]$var27_t0<-dbn.impute[[i]]$var27_t1;

dbn.impute[[i+1]]$var1_t1=as.numeric(dbn_dis24_t1_1);

dbn.impute[[i+1]]$var2_t1=as.numeric(dbn_dis24_t1_1);

dbn.impute[[i+1]]$var3_t1=as.numeric(dbn_dis24_t1_1);

dbn.impute[[i+1]]$var4_t1=as.numeric(dbn_dis24_t1_1);

dbn.impute[[i+1]]$var5_t1=as.numeric(dbn_dis24_t1_1);

dbn.impute[[i+1]]$var6_t1=as.numeric(dbn_dis24_t1_1);

dbn.impute[[i+1]]$var8_t1=as.numeric(dbn_dis24_t1_1);

dbn.impute[[i+1]]$var9_t1=as.numeric(dbn_dis24_t1_1);

dbn.impute[[i+1]]$var10_t1=as.numeric(dbn_dis24_t1_1);

dbn.impute[[i+1]]$var12_t1=as.numeric(dbn_dis24_t1_1);

dbn.impute[[i+1]]$var13_t1=as.numeric(dbn_dis24_t1_1);

dbn.impute[[i+1]]$var14_t1=as.numeric(dbn_dis24_t1_1);

dbn.impute[[i+1]]$var15_t1=as.numeric(dbn_dis24_t1_1);

dbn.impute[[i+1]]$var16_t1=as.numeric(dbn_dis24_t1_1);

dbn.impute[[i+1]]$var17_t1=as.numeric(dbn_dis24_t1_1);

dbn.impute[[i+1]]$var18_t1=as.numeric(dbn_dis24_t1_1);

dbn.impute[[i+1]]$var26_t1=as.numeric(dbn_dis24_t1_1);

dbn.impute[[i+1]]$var27_t1=as.numeric(dbn_dis24_t1_1);

dbn.impute[[i+1]]<-impute(pc.fit,dbn.impute[[i+1]])

}

data6<-dbn.impute[[6]] #data6 includes 24-hour predicted values
